# Short-Term Effects of Fine Particulate Matter and Temperature on Lung Function among Healthy College Students in Wuhan, China

**DOI:** 10.3390/ijerph120707777

**Published:** 2015-07-10

**Authors:** Yunquan Zhang, Mingquan He, Simin Wu, Yaohui Zhu, Suqing Wang, Masayuki Shima, Kenji Tamura, Lu Ma

**Affiliations:** 1 Department of Epidemiology and Health Statistics, School of Public Health, Wuhan University, Wuhan 430071, China; E-Mails: Yun-quanZhang@whu.edu.cn (Y.Z.); zyq7547182@163.com (M.H.); wusiminminmin@gmail.com (S.W.); 13349888239@163.com (Y.Z.); 2 Department of Nutrition and Food Hygiene, School of Public Health, Wuhan University, Wuhan 430071, China; E-Mail: swang2099@163.com; 3 Department of Public Health, Hyogo College of Medicine, Nishinomiya, Hyogo 663-8501, Japan; E-Mail: shima-m@hyo-med.ac.jp; 4 Environmental Health Sciences Division and Integrated Health Risk Assessment Section, National Institute for Environmental Studies, Tsukuba, Ibaraki 305-8506, Japan; E-Mail: ktamura@nies.go.jp

**Keywords:** college students, particulate matter, temperature, lung function, GEE

## Abstract

Ambient fine particulate matter (PM) has been associated with impaired lung function, but the effect of temperature on lung function and the potential interaction effect between PM and temperature remain uncertain. To estimate the short-term effects of PM_2.5_ combined with temperature on lung function, we measured the daily peak expiratory flow (PEF) in a panel of 37 healthy college students in four different seasons. Meanwhile, we also monitored daily concentrations of indoor and outdoor PM_2.5_ (particulate matter with an aerodynamic diameter ≤2.5 μm), ambient temperature and relative humidity of the study area, where the study participants lived and attended school. Associations of air pollutants and temperature with lung function were assessed by generalized estimating equations (GEEs). A 10 μg/m^3^ increase of indoor PM_2.5_ was associated with a change of −2.09 L/min in evening PEF (95%*CI*: −3.73 L/min–−0.51 L/min) after adjusting for season, height, gender, temperature and relative humidity. The changes of −2.17 L/min (95%*CI*: −3.81 L/min– −0.52 L/min) and −2.18 L/min (95%*CI*: −3.96 L/min–−0.41 L/min) in evening PEF were also observed after adjusting for outdoor SO_2_ and NO_2_ measured by Environmental Monitoring Center 3 kilometers away, respectively. An increase in ambient temperature was found to be associated with a decrease in lung function and our results revealed a small but significant antagonistic interactive effect between PM_2.5_ and temperature. Our findings suggest that ambient PM_2.5_ has an acute adverse effect on lung function in young healthy adults, and that temperature also plays an important role.

## 1. Introduction

Numerous studies have revealed that ambient particulate matter (PM) is associated with a range of adverse acute effects, including increased morbidity and mortality of cardiovascular and respiratory diseases [1,2,3], and with increased premature death and loss of life expectancy [4,5]. A recent systematic analysis for the Global Burden of Disease Study 2010 reported that ambient PM pollution accounted for 3.1 million (2.7 million to 3.5 million) deaths and 3.1% (2.7–3.4) of global Disability Adjusted of Life Years in 2010 [6]. Reduced lung function of both susceptible and healthy populations have also been related to short-term exposure to ambient PM [7,8,9,10]. Among the total suspended particles with different size fractions, particulate matter with an aerodynamic diameter ≤2.5 μm (PM_2.5_) has been found to be more responsible for adverse cardiopulmonary outcomes [9]. Moreover, based on consistent evidence for an association between long-term exposure to PM_2.5_ and lung cancer incidence or mortality, the International Agency for Research on Cancer has classified PM from outdoor air pollution as a class I carcinogen to humans [11].

It has been reported that ambient temperature and seasonal variation were both potential effect modifiers of the association between PM and all-cause, cardiovascular and respiratory mortality [12,13,14,15]. Several studies found that ambient temperature and PM may synergistically increase daily mortality [16,17,18,19,20], which indicated that higher temperatures would increase the mortality risk associated with PM. However, the effect of temperature on lung function has not been defined. Two recent studies suggested that higher ambient temperature was associated with lower lung function among both children with asthma [21] and young healthy university students [22]. Wu *et al.* [22] also pointed out that PM and temperature may synergistically weaken the lung function. However, to date, the relevant epidemiological evidence has been very limited, and it is still uncertain whether and how ambient temperature affects lung function among healthy adults.

In order to analyze the acute effects of ambient temperature and PM_2.5_ on lung function among healthy individuals, the present study followed a panel of 37 young healthy college students in four sequential seasons in Wuhan, China, and attempted to examine the effects of PM_2.5_ and temperature on lung function.

## 2. Objects and Methods

### 2.1. Study Participants and Design

We recruited a panel of 37 healthy, non-smoking college students (18 male students and 19 female students) within one class from the medical school of Wuhan University in October 2009. Students were between the ages of 19 and 21, and were free of cardiovascular, pulmonary and other chronic diseases. The study was approved by the ethics committee at Wuhan University and the written informed consent was provided by all study participants.

According to the climate characteristics and seasonal variation of Wuhan, the study was conducted in four seasons: 29 October 2009–11 November 2009 (autumn), 23 December 2009–5 January 2010 (winter), 24 March 2010–6 April 2010 (spring) and 24 July 2010–6 August 2010 (summer). Each study period lasted 14 days in each season.

### 2.2. Lung Function Testing

Spirometry test training was conducted by trained field technicians among the participants before the study. Every Electronic PEF Diary (Vitalograph Ltd., Buckingham, UK) was adjusted by a calibrated 3 L Precision Syringe (Vitalograph Ltd.) before each period of study. Morning (07:30) and evening (22:30) PEF were measured daily by the participants themselves with the Electronic PEF Diary during the study. Each PEF value was measured two to five times, and the error of every two measurements was required to be within 10% before being recorded as a valid data point.

### 2.3. Environmental Measurements

PM_2.5_ mass was collected on fiberglass filters by four small hand-holding personal samplers, each of which was set 1.5 m high both inside and outside of the male and female dormitories. The sampler, which consisted of a particle diameter of cutter (ATPS-20H, Sibata Scientific Technology Inc., Tokyo, Japan) and an air pump (MP-∑3, Sibata Scientific Technology Inc.), consecutively collected PM samples at a constant flow rate of 1.5 L/min for 24 h starting from 7:30 a.m..

The PM_2.5_ mass concentrations were obtained by weighing the filters before and after the sampling, always after a storage period (24 h) in a temperature- and humidity-controlled room (ambient temperature, 23 ± 0.2 °C; relative humidity, 50% ± 1%), using an ultra-microbalance with a sensitivity of 0.1 μg (UMX-2, Mettler-Toledo Inc., Columbus, OH, USA).

4 HOBO Pro V2 loggers (Onset Corp., Pocasset, MA, USA) were used for real-time temperature and relative humidity measurements at 1-min intervals in/out dormitories. Instruments were calibrated according to manufacturer’s specifications in advance.

24 h average concentrations of gaseous pollutants including sulfur dioxide (SO_2_) and nitrogen dioxide (NO_2_) were obtained from the Liyuan site of Wuhan Environmental Monitoring Center (WEMC), located 3 km away from the medical campus.

### 2.4. Statistical Analysis

During each period, real-time levels of indoor/outdoor environmental variables were aggregated as 24 h averages, sequentially followed by matching with morning and evening PEF data prior to analysis. Data pertaining to air pollutants, meteorological factors and measurements of PEF were initially examined using descriptive summary statistics and Bivariate-Spearman’s correlations. Multivariate associations were assessed using statistical models of generalized estimating equations (GEEs).

Single-pollutant and two-pollutant models (PM_2.5_-SO_2_ & PM_2.5_-NO_2_) were used to estimate the effects of indoor/outdoor PM_2.5_ and temperature on morning/evening PEF. The following covariates, selected based on practices used by many previous epidemiological studies, were included in single-pollutant model: gender (categorical), height, season (categorical), 24 h average temperature and relative humidity. SO_2_ or NO_2_ was also adjusted for in the two-pollutant models.

Both single-pollutant and two-pollutant models investigated lagged exposures: exposure measured on the day of spirometry test (lag 0), 1 day prior to spirometry test (lag 1), 2 days prior to spirometry test (lag 2), the average of exposures measured 1 day prior to and on the day of spirometry test (lag 0–1), and the average of exposures measured 2 days prior, 1 day prior and on the day of spirometry test (lag 0–2).

To explore the interaction between PM_2.5_ and temperature, a multiplicative interaction term was included along with terms for main effects in above PM_2.5_-temperature joint models [22]. Final results were reported as changes of PEF (L/min) with 95% confidence intervals associated with 10 μg/m^3^ increases of PM_2.5_ and 1 °C increase of temperature. All analyses were performed using SPSS software version 17.0 (SPSS Inc., Chicago, IL, USA) and significance level was set at *p* < 0.05 (two-tailed).

## 3. Results

### 3.1. Descriptive Statistics of Health and Exposure Data

The study subjects were 18 male and 19 female college students aged 21.7 (SD 0.8) years. During the entire study periods, these subjects spent most of their time indoors, including in the dormitories (more than 10 h), library and classrooms, and their average outdoor time was 1.7 h/day (7.1%). A total of 3538 person-times of valid PEF data (85.4%) were obtained compared to the 4144 expected. Of this, 1758 of the expected 2072 person-times of morning PEF (84.8%) and 1780 of the expected 2072 person-times of evening PEF (85.9%) were obtained. The mean morning and evening PEF (standard deviation) were 580.2(98.9) L/min and 582.9(94.9) L/min, respectively, in males, 378.1(53.8) L/min and 381.6(50.2) L/min, respectively, in females. The detailed characteristics and PEF measures of the study subjects are presented in Table 1.

**Figure 1:**
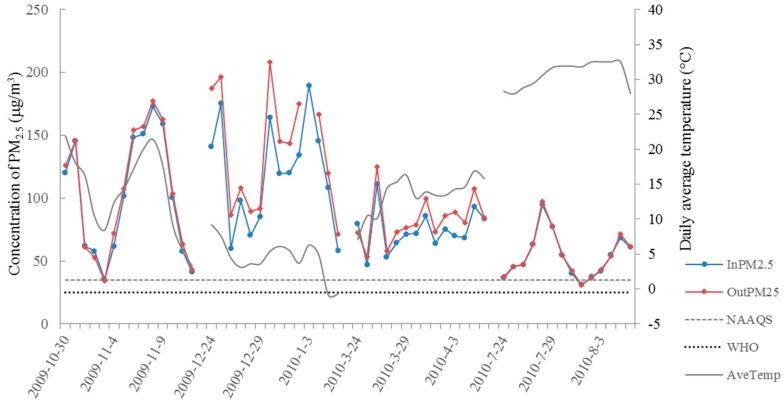
Daily indoor/outdoor PM_2.5_ concentration and average temperature during study period. In PM2.5, daily average concentration of indoor PM_2.5_; Out PM25, daily average concentration of outdoor PM_2.5_; NAAQS, National Ambient Air Quality Standard for PM_2.5_ in U.S.; WHO, World Health Organization air quality guideline for PM_2.5_; PM_2.5_, particulate matter <2.5 µm in aerodynamic diameter.

Daily air pollution levels and meteorological conditions during the four study periods are presented in Table 2. Variation in the trend of indoor/outdoor PM_2.5_ and daily average temperature is shown in Figure 1. Concentrations of indoor/outdoor PM_2.5_ were highest in winter and lowest in summer, both of which were exceeding WHO guideline for PM_2.5_ (25 μg/m^3^).

**Table 1:** Basic characteristics and PEF measures of the study subjects.

Characteristics	Male	Female	All
	(*n* = 18)	(*n* = 19)	(*n* = 37)
Basic information of subjects			
Age (year) ^a^	22.0(0.9) ^b^	21.5(0.6)	21.7(0.8)
Height (cm)	171.7(6.0)	158.6(3.8)	164.9(8.3)
Weight (kg)	59.1(5.8)	50.6(5.8)	54.7(7.1)
Out time (hours/day) ^c^	1.2(0.9)	2.1(1.0)	1.7(1.1)
No. of PEF measurements			
N a.m.	46.9(10.1)	48.1(7.1)	47.5(8.9)
N p.m.	46.9(11.2)	49.3(6.4)	48.1(9.0)
Mean PEF (L/min)			
Morning PEF	580.2(98.9)	378.1(53.8)	475.2(128.1)
Evening PEF	582.9(94.9)	381.6(50.2)	477.0(125.3)

PEF, peak expiratory flow; ^a^ The age of subjects in October, 2010; ^b^ Mean and standard deviation; ^c^ Not recorded in summer.

**Table 2:** Descriptive statistics for the meteorological conditions and air pollutants.

	Spring (N = 14)			Summer (N = 14)			Autumn (N = 14)			Winter (N = 14)		
	Mean(sd)	Median	Min/Max	Mean(sd)	Median	Min/Max	Mean(sd)	Median	Min/Max	Mean(sd)	Median	Min/Max
Indoor												
PM_2.5_ (μg/m^3^)	74.1(16.2)	71.5	46.9/110.8	53.8(17.8)	50.8	31.1/94.5	91.3(43.7)	93.5	29.2/149.6	110.6(42.3)	112.1	49.3/184.2
Temperature (°C)	18.5(2.8)	19.1	14.2/22.7	33.9(2.5)	34.5	29.4/37.4	19.6(2.2)	19.5	16.3/23.0	11.4(0.8)	11.2	10.3/12.6
Humidity (%)	55.6(6.5)	54.5	45.7/69.1	64.3(7.8)	64.0	54.2/74.8	56.0(11.7)	55.3	36.0/75.2	62.4(6.3)	61.9	50.6/75.8
Outdoor												
PM_2.5_ (μg/m^3^) ^a^	82.6(18.7)	79.5	53.4/124.7	54.5(18.3)	50.5	31.0/97.4	104.2(49.4)	106.6	34.0/177.7	143.5(51.2)	144.9	71.2/288.9
Temperature (°C)	15.7(2.8)	16.0	9.6/20.4	33.3(1.9)	33.5	29.8/36.1	16.7(5.2)	18.3	7.0/22.4	7.8(2.5)	7.9	3.8/12.0
Humidity (%)	61.0(12.2)	58.2	44.2/82.4	66.4(6.3)	66.9	57.3/76.3	64.4(13.7)	66.8	38.0/80.0	55.7(10.2)	57.9	37.9/67.0
Liyuan ^b^												
PM_10_ (μg/m^3^)	112.7(38.9)	118.0	60.0/190.0	67.6(16.7)	66.0	43.0/102.0	94.4(54.9)	83.0	20.0/176.0	151.3(45.9)	161.0	52.0/240.0
SO_2_ (μg/m^3^)	39.1(18.9)	42.5	9.0/62.0	19.0(8.0)	19.0	5.0/30.0	39.7(21.8)	45.0	11.0/80.0	56.0(11.9)	58.0	26.0/80.0
NO_2_ (μg/m^3^)	41.9(12.8)	41.6	20.8/64.0	26.3(7.2)	25.6	14.4/41.6	50.0(26.2)	40.8	22.4/95.2	53.5(17.8)	50.4	22.4/86.4

PM_2.5_, particulate matter <2.5 µm in aerodynamic diameter; ^a^ The number of measurement of outdoor PM_2.5_ was 13 in winter; ^b^ Data was collected at the Liyuan site of WEMC.

### 3.2. Correlation Matrix between Environmental Variables

The Spearman correlation coefficients for pollutants and meteorological variables are presented in Table 3. Indoor PM_2.5_ concentration was highly correlated with outdoor PM_2.5_ (*r_s_* = 0.942). Indoor temperature was also highly correlated with outdoor temperature (*r_s_* = 0.921). According to data values for gaseous pollutants collected at the Liyuan site of WEMC, both indoor and outdoor PM_2.5_ were moderately correlated with SO_2_ and NO_2_.

**Table 3:** Spearman correlation coefficients for pollutants and meteorological variables.

	InPM_2.5_	SO_2_^a^	NO_2_^a^	InTemp.	OutTemp.	InRh (%)	OutRh (%)
OutPM_2.5_	0.942	0.589	0.759	−0.529	−0.406	0.202	−0.038
InPM_2.5_	—	0.505	0.693	−0.406	−0.260	0.245	0.046
SO_2_^a^	—	—	0.712	−0.571	−0.419	−0.138	−0.519
NO_2_^a^	—	—	—	−0.500	−0.326	−0.003	−0.248
InTemp.	—	—	—	—	0.921	0.256	0.354
OutTemp.	—	—	—		—	0.329	0.219
InRh (%)	—	—	—		—	—	0.612

InPM_2.5_, daily average concentration of indoor PM_2.5_; OutPM_2.5_, daily average concentration of outdoor PM_2.5_; InTemp., indoor daily average temperature; OutTemp., outdoor daily average temperature; InRh (%), indoor daily average relative humidity; OutRh (%), outdoor daily average relative humidity; PM_2.5_, particulate matter <2.5 µm in aerodynamic diameter. ^a^ Data of SO_2_ and NO_2_ was collected at the Liyuan site of WEMC.

### 3.3. Global and Seasonally Stratified Analysis of the Estimated Effects of PM_2.5_ and Temperature

#### 3.3.1. Global Analysis of the Effects of PM_2.5_ and Temperature on lag 0

Table 4 shows that a 10 μg/m^3^ increase of indoor PM_2.5_ on lag0 was associated with a change of −2.09 L/min in evening PEF (95%*CI*: −3.73–−0.51 L/min) adjusted for season, gender, height, temperature and relative humidity. After adjusting for gaseous pollutants (SO_2_/NO_2_), changes of −2.17 L/min (95%*CI*: −3.81–−0.52 L/min) and −2.18 L/min (95%*CI*: −3.96–−0.41 L/min), respectively in evening PEF were found in the two-pollutant models. Also, a 10 μg/m^3^ increase of outdoor PM_2.5_ was associated with a decrease of 1.54 L/min in evening PEF (95%*CI*: −3.03–−0.04 L/min). Besides, no consistent adverse impacts of SO_2_ and NO_2_ on PEF were observed in our study.

Additionally, in most models, a significant and negative association was consistently observed between both indoor/outdoor temperature and morning/evening PEF. In the single-pollutant model, for instance, a 1 °C increase in indoor temperature on lag 0 was associated with a decrease of 1.58 L/min (95%*CI*: −2.86–−0.30 L/min) and 2.16 L/min (95%*CI*: −3.67–−0.65 L/min) in morning and evening PEF, respectively; and a 1 °C increase in outdoor temperature on lag0 was associated with a decrease of 0.84 L/min (95%*CI*: −1.63–−0.04 L/min) and 1.22 L/min (95%*CI*: −2.22–−0.22 L/min) in morning and evening PEF, respectively. Moreover, the interactive effects between temperature and PM_2.5_ on evening PEF were found to be significantly positive, in spite of the small interactive effects compared to the main effects. Specifically, an increase in temperature reduced the adverse effect of PM_2.5_ on lung function, and an increase in concentration of PM_2.5_ also weakened the association between temperature and lung function.

**Table 4:** Global analysis of the effects of PM_2.5_, temperature and interaction term on PEF.

	Morning PEF						Evening PEF					
	Indoor			Outdoor			Indoor			Outdoor		
	β	95%*CI*	*P*	β	95%*CI*	*P*	β	95%*CI*	*P*	β	95%*CI*	*P*
Single-pollutant model												
PM_2.5_, 10 μg/m^3^	−1.08	(−2.95,0.80)	0.262	0.07	(−1.44,1.57)	0.929	−2.09	(−3.73,−0.51)	0.015	−1.29	(−2.75,0.17)	0.084
Temp., 1 °C	−1.58	(−2.86,−0.30)	0.016	−0.84	(−1.63,−0.04)	0.039	−2.16	(−3.67,−0.65)	0.005	−1.22	(−2.22,−0.22)	0.017
PM_2.5_*Temp.	0.00	(−0.01,0.01)	0.598	0.00	(−0.01,0.01)	0.968	0.01	(0.00,0.02)	0.033	0.01	(0.00,0.02)	0.043
Two-pollutant model (SO_2_)												
SO_2_, 10 μg/m^3^	−0.12	(−1.41,0.17)	0.851	0.31	(−1.11,1.72)	0.670	0.74	(−0.10,1.59)	0.086	1.14	(−0.29,2.57)	0.205
PM_2.5_, 10 μg/m^3^	−1.05	(−2.96,0.85)	0.279	−0.01	(−1.51,1.48)	0.987	−2.17	(−3.81,−0.52)	0.010	−1.27	(−2.73,0.18)	0.087
Temp., 1 °C	−1.60	(−2.93,−0.26)	0.019	−0.83	(−1.63,−0.03)	0.043	−2.12	(−3.62,−0.63)	0.005	−1.27	(−2.28,−0.26)	0.014
PM_2.5_*Temp.	0.00	(−0.01,0.02)	0.592	0.00	(−0.01,0.01)	0.967	0.01	(0.00,0.02)	0.038	0.01	(0.00,0.02)	0.067
Two-pollutant model (NO_2_)												
NO_2_, 10 μg/m^3^	0.41	(−1.20,2.02)	0.619	1.01	(−0.65,2.67)	0.232	0.25	(−1.12,1.61)	0.724	1.17	(−0.44,2.79)	0.153
PM_2.5_, 10 μg/m^3^	−1.23	(−3.24,0.77)	0.228	−0.34	(−1.99,1.31)	0.686	−2.18	(−3.96,−0.41)	0.016	−1.54	(−3.03,−0.04)	0.044
Temp., 1 °C	−1.51	(−2.86,−0.16)	0.029	−0.79	(−1.61,0.03)	0.060	−2.15	(−3.67,0.63)	0.006	−1.34	(−2.37,−0.30)	0.011
PM_2.5_*Temp.	0.00	(−0.01,0.01)	0.613	0.00	(−0.01,0.01)	0.979	0.01	(0.00,0.02)	0.033	0.01	(0.00,0.02)	0.058

Generalized estimating equations were adjusted for season, gender, height, relative humidity. PEF, peak expiratory flow; Temp., daily average temperature; PM_2.5_*Temp., interaction term between PM_2.5_ and Temp.; PM_2.5_, particulate matter <2.5 µm in aerodynamic diameter.

#### 3.3.2. Lagged and Cumulative Effects of PM_2.5_ and Temperature on PEF

**Figure 2:**
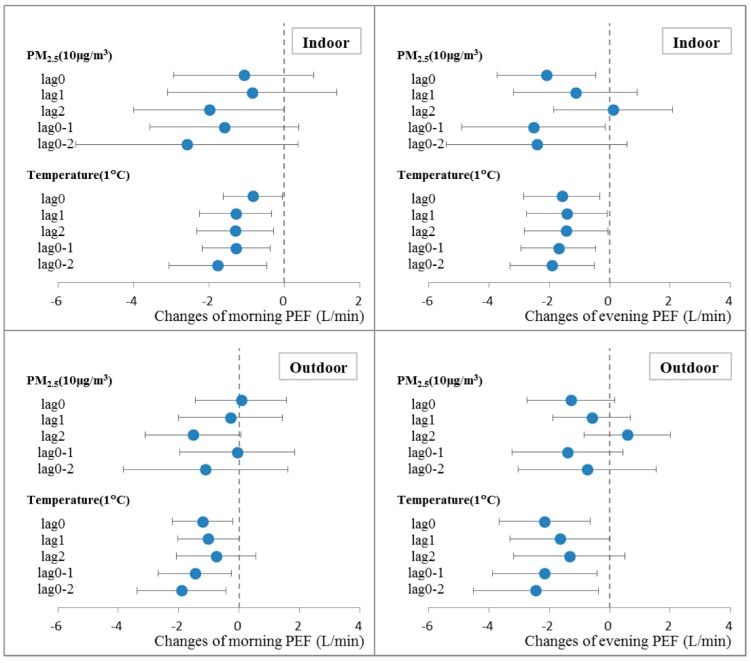
Lagged and cumulative effects of indoor/outdoor PM_2.5_ and temperature on morning/evening PEF up to two days in single-pollutant model. Estimates for changes in PEF with 95% confidence intervals were shown per 10 μg/m^3^ increase of PM_2.5_ or 1 °C increase of temperature, which were adjusted for season, gender, height, temperature and relative humidity.

Figure 2 shows that the associations between PM_2.5_ and lung function varied by days of lag. For morning PEF, lag 2 PM_2.5_ showed the largest impact. For evening PEF, stronger associations were observed on days of lag 0 and lag 0–1. Higher temperatures were consistently found to be associated with lower PEF on various lags. Moreover, cumulative lags (lag 0–1 and lag 0–2) of temperature were more strongly associated with both morning PEF and evening PEF than single day of lags.

#### 3.3.3. Seasonally Stratified Analysis of the Effects of PM_2.5_ and Temperature

The seasonally stratified effects of outdoor PM_2.5_ on current morning/evening PEF (Table 5) revealed no significant associations in the seasons of spring, summer and autumn. In winter, however, a 10 μg/m^3^ increase of outdoor PM_2.5_ was associated with a change of −1.28 L/min (95%*CI*: −2.50–−0.06 L/min) in evening PEF. Of note, a 1 °C increase of outdoor temperature was found to be significantly associated with an increase of 2.07 L/min (95%*CI*: 0.34–3.80 L/min) for evening PEF in winter, which was not consistent with the global analysis in Table 4. Analysis of seasonal interactions between PM_2.5_ and temperature was not informative because of wide confidence intervals, likely resulting from short duration of examination in each season.

**Table 5:** Seasonally stratified analysis of effects of outdoor PM_2.5_ and temperature on PEF of the current day.

Variables	Morning PEF			Evening PEF		
	β	95%*CI*	*P*	β	95%*CI*	*P*
Spring						
PM_2.5_, 10 μg/m^3^	0.53	(−0.91,1.96)	0.472	−0.81	(−3.11,1.49)	0.490
Temp., 1 °C	−0.44	(−1.65,0.77)	0.477	−0.46	(−1.59,0.68)	0.430
Summer						
PM_2.5_, 10 μg/m^3^	−1.77	(−4.51,0.97)	0.204	−1.14	(−4.11,1.83)	0.452
Temp.,1 °C	−7.75	(−21.66,6.16)	0.275	1.66	(−7.58,10.91)	0.724
Autumn						
PM_2.5_, 10 μg/m^3^	0.30	(−0.77,1.37)	0.585	0.62	(−0.40,1.65)	0.233
Temp., 1 °C	−1.01	(−1.88,−0.13)	0.024	−0.93	(−1.89,0.04)	0.060
Winter						
PM_2.5_, 10 μg/m^3^	−0.40	(−2.08,1.28)	0.639	**−1.28**	**(−2.50,−0.06)**	**0.040**
Temp., 1 °C	−0.24	(−2.95,2.48)	0.865	**2.07**	**(0.34,3.80)**	**0.019**

Generalized estimating equations were adjusted for gender, height, relative humidity. PEF, peak expiratory flow; Temp., daily average temperature; PM_2.5_, particulate matter <2.5 µm in aerodynamic diameter.

## 4. Discussion

The present study followed a panel of 37 young healthy college students in four various study periods, and assessed the effects of daily average indoor/outdoor PM_2.5_ and ambient temperature on morning/evening PEF simultaneously. Our results revealed that increased PM_2.5_ was associated with reduced PEF. Of note, the limitation in the two-pollutant models was that SO_2_ and NO_2_ data was available only at a site 3 km away, hence errors arising from the heterogeneous and differential exposure measurements could weaken the inferred results from co-pollutant models. Also, in the global analysis, higher ambient temperature was found to be associated with lower PEF. However, higher outdoor temperature showed a significant protective effect on evening PEF in winter. In addition, we also observed a very small antagonistic effect between temperature and PM_2.5_, which attenuated the harmful impacts of temperature and PM_2.5_. These findings may have helpful implications for us to better understand the adverse pulmonary impact of exposure to PM_2.5_ and the association between temperature and lung function among healthy adults.

### 4.1. Effects of PM_2.5_ on Lung Function

Previous studies have demonstrated that an elevated concentration of PM_2.5_ was associated with decreased lung function in susceptible populations, including children, the elderly, and those with respiratory conditions such as asthma and chronic obstructive pulmonary disease (COPD) [10,23,24,25,26]. Subjects of the present study were young and healthy college students who might be more resistant to atmospheric pollution than the susceptible populations [27]. The negative association between PM_2.5_ and PEF was significant, and so were the lagged and cumulative effects.

The present study revealed a stronger association between indoor/outdoor PM_2.5_ and evening PEF than morning PEF on the current day. Increased indoor/outdoor PM_2.5_ concentration was significantly associated with a decrement in current evening PEF but not the morning PEF, and larger effect estimates were also observed in the evening rather than in the morning (Table 4). Several previous studies have examined the associations between PM and morning/evening PEF and showed inconsistent results. A panel study in children with chronic respiratory symptoms in Finland revealed that the changes in morning and evening PEF for the inter-quartile range (14 μg/m^3^) of PM_2.5_ on the previous day were −1.06 L/min (*p* < 0.05) and −0.43 L/min (not significant), respectively [28]. Another study in European countries found that an increase of 10 μg/m^3^ in PM_10_ was associated with PEF changes of 0.01 L/min (N.S.) in the morning and −0.06 L/min (*p* < 0.05) in the evening, respectively [29]. In 2011, Yamazaki *et al.* [10] studied the effect of hourly concentration of PM on PEF in hospitalized children in Japan and no difference was found in the effect of PM on morning/evening PEF. Thus, it was still not clear whether exposure to PM was more strongly associated with morning or with evening lung function.

Both indoor and outdoor PM_2.5_ were associated with PEF among the college students (Table 4 & Figure 2), which might result from the high correlation and strong permeation from the outdoor environment. In two previous studies conducted in Japan [30] and America [31], exposure to indoor PM_2.5_ showed a stronger association with decreased lung function when compared with outdoor PM_2.5_. Notably, mean concentration of indoor PM in these two studies was higher than that of outdoor, and indoor and outdoor PM_2.5_ were weakly correlated. There was evidence showing that indoor-generated particles might be more bioactive than outdoor particles by assessing the *in vitro* toxicity of indoor and outdoor PM_2.5_ collected in Boston-area homes [32]. Even in the absence of obvious pollutant sources, some allergens, such as house dust mites, might also contaminate the indoor environment. In our study, college students spent most of their time in the dormitories and classrooms on campus. Therefore, assessment of the indoor micro-environment was more appropriate to evaluate the association between PM and PEF of subjects for the present study. Important strength of this study is that ambient PM_2.5_ exposure was monitored outdoors and indoors where study participants lived and attended school, resulting in more accurate exposure estimates than that based on PM_2.5_ measured at central site monitors.

### 4.2. Effects of Temperature on Lung Function

Several previous studies have reported that seasonal differences in temperature were associated with lung function, and warmer temperature led to lower FEV_1_ in cystic fibrosis patients [19] and lower PEF in subjects with chronic pulmonary diseases [33]. A recent panel study of 270 asthmatic children in five cities in Australia revealed that higher ambient temperature was significantly associated with lower PEF and FEV_1_ even after controlling for children’s respiratory symptoms and air pollutants (PM_2.5_ and other gaseous pollutants), and the effects of temperature on children’s lung function varied by cities [21].

It was also demonstrated in our research that higher indoor/outdoor temperature was associated with lower morning/evening PEF among college students in the global analysis. Results of seasonally stratified analyses suggested that, however, in the winter with general low average temperatures (7.8 ± 2.5 °C), a 1 °C increase of outdoor temperature was associated with an increase of PEF by 2.07 L/min (95%*CI*: 0.34–3.80 L/min), which was contradictory to the global analysis and the studies referred to above. Moreover, significant positive associations between temperature and lung function were found in several epidemiological studies. A previous long-term study of 76 elderly COPD patients conducted in east London found that a fall in outdoor or bedroom temperature was associated with increased frequency of exacerbation and decline in lung function [20]. Belli *et al.* [34] reported that lower outdoor temperatures were associated with increased symptom severity and reduced lung function in former smokers with COPD during cold season as well. As for children with asthma, adverse effects of low indoor temperatures on lung function were also found in the Heating House and Health Study conducted in New Zealand [35].

As many studies indicated, both extremely low and high temperatures were robustly associated with adverse cardiopulmonary and cardiovascular events, including increased morbidity and mortality [13,36,37,38,39]. The authors further speculated that ambient temperature could be a potential and important confounding factor for lung function. Nevertheless, health effects of ambient temperature might vary by seasonal and climatic factors, study populations and study locations [21,37,38,40,41,42].

Very few studies have discussed the possible mechanisms underlying the inverse association between both high/low temperature and lung function, which remained unclear. For susceptible populations, such as asthmatic children, one possible explanation is that higher temperature is associated with higher allergen exposure such as pollen loads that may potentially lead to trigger of asthma [21], and infectious agents (such as through *P. aeruginosa*) that may mediate the association between temperature and lung function among patients with cystic fibrosis lung disease [19]. Another possibility might be that higher temperature is associated with airway drying, which may result in bronchoconstriction and decrement of lung function [43]. Besides, the reduction of lung function can be caused by increased airway inflammation in cryogenic environment among both asthmatic and COPD patients. On the other hand, cold temperatures can induce peripheral vasoconstriction and shunt blood centrally, and inhalation of cold air can also cause post-exertional bronchoconstriction in asthmatics, both of which thus will result in decreased lung function [20].

### 4.3. Interactive Effects between PM_2.5_ and Temperature on Lung Function

Seasonal variations have been reported in a few previous studies about the effects of ambient PM on non-accidental mortality of the entire population [12,15,44,45]. Another two studies in Europe revealed that season and temperature levels strongly modified the PM_10_-mortality association [14,15]. Synergistic effects of PM_10_ and high temperature on daily non-accidental, cardiovascular, and cardiopulmonary mortality were also found in a Chinese study conducted in Wuhan [17]. However, very few studies have explored the interactive effects of PM and ambient temperature on human lung function.

In our research, both ambient PM_2.5_ and temperature demonstrated a certain seasonal variation in the effects on lung function of healthy college students. Moreover, PM_2.5_ and temperature were found to have a significant antagonistic interactive effect in reducing PEF (Table 4), which was not consistent with a recent study conducted in Beijing of China [22]. However, the effect size of interaction was very small compared to the main effects, so it is hard to say whether the interactive effect between PM_2.5_ and temperature is of clinical significance for young and healthy adults in the present study.

The seasonal variation of effects of PM on PEF might be explained by changes in PM sources and constituents with different toxicological characteristics in each season [45,46], and various chemical components of ambient PM_2.5_ may play different and complicated roles in affecting lung function of young healthy adults [9]. However, to date, the mechanisms underlying the interactive effects of ambient PM pollution and temperature on lung function are still unclear. It was biologically assumed that changes of environmental temperature act on the thermoregulatory system, activation of which has direct or indirect effects on the entry of toxicants into the body, thus enhancing or attenuating total intake of airborne pollutants [14,47]. Another possibility might be that through effects on reaction kinetics, higher daily ambient temperatures lead to increased ozone levels [48], which can also result in impaired lung function. The confounding effect of ozone associated with temperature may finally have modified the observed adverse impact of PM on lung function. In addition, some other potential factors may affect both air pollutants and temperature [49], thus resulting in interactive effects between pollutants and temperature.

## 5. Conclusions

In conclusion, our research suggests that exposure to high concentration of PM_2.5_ has an acute adverse effect on lung function among young healthy adults in Wuhan of China. After adjusting for season and other confounding factors, temperature was also found to be associated with lung function, and temperature can modify the effect of PM_2.5_ on lung function.
